# Evaluation of the mir-126, mir-182, and mir-486-5p Expression Signature of Head and Neck Squamous Cell Carcinomas and Lung Squamous Cell Carcinomas

**DOI:** 10.5146/tjpath.2021.01528

**Published:** 2021-05-15

**Authors:** Gizem Issın, Zafer Kucukodacı, Ismail Yılmaz, Evren Erkul, Ersin Tural, Dilaver Demirel, Atila Gungor, Sukru Yıldırım

**Affiliations:** Department of Pathology, Erzincan Binali Yildirim University, Mengucek Gazi Training and Research Hospital, Erzincan, Turkey; University of Health Sciences, İstanbul Sultan 2. Abdülhamid Han Training Hospital, Istanbul, Turkey; Department of Otorhinolaryngology, Gulhane Medical School, University of Health Sciences, İstanbul Sultan 2. Abdülhamid Han Training Hospital, Istanbul, Turkey; Department of Pediatrics, University of Health Sciences, İstanbul Sultan 2. Abdülhamid Han Training Hospital, Istanbul, Turkey; Department of Pathology, University of Health Sciences, Gaziosmanpasa-Taksim Health Application and Research Center, Istanbul, Turkey; Department of Otorhinolaryngology, Medical Park Goztepe Hospital, Istanbul, Turkey; Department of Pathology, Maltepe University, Faculty of Medicine, Istanbul, Turkey

**Keywords:** Head and neck, Lung, Squamous cell carcinoma, mir-126, mir-182, mir-486-5p

## Abstract

***Objective:*** Although squamous cell carcinomas (SCCs) originating from different anatomic localizations display a similar histological appearance under light microscopy, they may differ in terms of epigenetic and genetic features. The aim of this study was to analyze mir-126, mir-182, and mir-486-5p expression levels in head and neck SCCs and lung SCCs, and to identify localization-specific miRNA expression profiles.

***Material and Method:*** The expression levels of mir-126, mir-182, and mir-486-5p were analyzed in lung, oral cavity, laryngeal, and hypopharyngeal SCCs in 40 patients, using quantitative real-time polymerase chain reaction.

***Results:*** The findings showed that lung, oral cavity, laryngeal, and hypopharyngeal SCCs have distinct mir-126 and mir-486-5p expression profiles. It was also observed that mir-126 and mir-486-5p expression levels were highly specific to the tumor localization.

***Conclusion:*** These findings highlighted that SCCs originating from different anatomic localizations have different miRNA expression profiles. miRNA expression analysis can be used to predict the primary localizations of those SCCs.

## INTRODUCTION

Squamous cell carcinoma (SCC) is a tumor that originates from mucosal or epidermal keratinocytes and can occur in different localizations such as the head and neck, lung, cervix, and skin (1). SCCs arising from different anatomic localizations often exhibit a similar histological appearance under the light microscope and this may cause diagnostic challenges in some cases, especially in the differential diagnosis of SCC masses in the lungs of patients with head and neck squamous cell carcinoma (HNSCC) (2–4).

The epithelium overlying the upper aerodigestive tract is continuous with the respiratory epithelium, so carcinogens such as tobacco products may show similar carcinogenic processes along the upper aerodigestive and respiratory tracts at the same time. Therefore, a second primary tumor may develop in the lungs of patients with HNSCC (5). In addition, the lung is the most common site of visceral metastases in these patients (6). Histopathological examination often does not provide enough insight to suggest the exact localization of the primary anatomic region of the tumor. Therefore, differential diagnosis between the second primary lung tumor and a lung metastasis is challenging in patients with HNSCC. Although SCCs that arise from different anatomic localization have a similar histopathological appearance, tumors may differ in terms of genetic and epigenetic features, such as the miRNA expression profile, which are specific to the anatomic localization (7–10).

miRNAs, which are single-stranded non-protein-coding RNAs generally 18–24 nucleotides long, are capable of controlling gene expression at the post-transcriptional level (11). Studies have revealed that miRNAs in humans have an essential function in regulating various biological pathways, such as the cell cycle, proliferation, development, and growth (12). Dysregulation in the expression profile of miRNAs disrupts biofeedback and gene expression control mechanisms, which may lead to the development of cancer (13). Several studies have revealed that miRNAs show different expression profiles according to tissue and tumor types (14,15). This highly specific expression profile can be used for diagnostic purposes, such as the discrimination of primary cancers and their metastases (15).

Research based on the integrated analysis of The Cancer Genome Atlas (TCGA) database data has revealed that mir-126, mir-182 and mir-486-5p were the consistently and significantly dysregulated miRNAs in lung cancer (16–19). Research has also indicated that profile analysis of this miRNA expression could differentiate lung carcinoma patients from healthy tissue or patients with lung metastasis of tumor (20–25). In this study, mir-126, mir-486-5p and mir-182 expression levels were analyzed in lung, oral cavity, hypopharynx, and laryngeal SCC, and evaluations were made of the association of tumor anatomic localization with the mir-126, mir-486-5p, and mir-182 expression profile.

## MATERIAL and METHODS

### Patient Selection

A total of 40 subjects were enrolled from patients who underwent surgical resection for oral cavity, laryngeal, hypopharyngeal, or lung SCC between January 2012 and December 2015 in the Department of Thoracic and Otorhinolaryngology Surgery, 2nd Sultan Abdulhamid Han Training and Research Hospital. Analyses were conducted on the formalin-fixed, paraffin-embedded tissues from 40 cases comprising five each from glottic and supraglottic laryngeal SCC cases, and 10 each from oral cavity, hypopharyngeal, and lung SCC cases. None of the patients had a known history of surgery for recurrent cancer, nor had they received neoadjuvant chemo/radiotherapy. Clinical data were gathered from all patients. The details of the clinical and pathological data are presented in Table 1. Approval for the study was granted by the Local Medical Ethics Committee (approval No. 29.01.2016/1491-17-16/1539). All study procedures were performed according to the Declaration of Helsinki principles.

**Table 1:** Clinicopathological characteristics of the patients in this study.

**Clinical features**	**Lung SCC**	**Oral SCC**	**Hypopharyngeal SCC**	**Laryngeal SCC**
Mean age (max-min)	70 (61-77)	69 (34-87)	62 (54-75)	62 (46-73)
Gender				
Male (n)	5	5	5	5
Female (n)	4	5	5	4
Histological type				
Keratinizing type SCC	5	5	5	5
Non-keratinizing type SCC	4	5	5	4
Histological grade				
Grade I Well-differentiated	-	-	-	-
Grade II Moderately-differentiated	8	8	9	9
Grade III Poorly-differentiated	1	2	1	
Tumor stage				
Stage-III (n)	9	5	5	5
Stage-IV (n)		5	5	4
Overall Survival (month) mean ± S.E.	67 ± 11.2	42 ± 9.5	62 ± 17.4	59 ± 12.5
5-year Survival rates	44.4%	30%	40%	44.4%

**SCC:** Squamous cell carcinoma

### Selection of miRNA

The selection of miRNA was based on a literature review of the research based on the integrated analysis of the Cancer Genome Atlas (TCGA) database data of miRNA expression profiles in lung SCCs (16–25). To identify miRNAs with a predictive potential to differentiate lung SCCs and HNSCCs, the research focused on miRNAs that play a critical role in lung carcinogenesis, lung tissue, or tumor specificity. miRNAs accepted as oncoMİR (e.g., miR-21, mir-30) and miRNAs whose expression levels were highly associated with squamous epithelia-containing tissues, such as mir-205, mir-31 and mir-203, were not selected (26–29). Three miRNAs (mir-126, mir-182 and mir-486) that could differentiate lung carcinoma patients from healthy tissue or patients with lung metastasis were selected for the study.

### RNA Extraction from Tissue Samples and Quantitative Real-Time Polymerase Chain Reaction (PCR) of miRNAs

The slides of each patient were re-examined to determine the areas where tumor cells were most dense. Tumors and corresponding non-neoplastic squamous epithelium were manually micro-dissected from 5μm- thick paraffin sections and placed in 1.5 mL microcentrifuge tubes. After deparaffinization and rehydration, total RNA was isolated using Recover All Total Nucleic Acid Isolation Kit for FFPE tissue in accordance with the manufacturer’s instructions. The RNA concentration was measured with a NanoDrop 1000 Spectrophotometer (Thermo Scientific, USA). Ten nanograms of total RNA were reverse-transcribed for RNU6b, mir-126, mir-182, and mir-486-5p using the Taqman MicroRna Reverse Transcription Kit. Reverse transcription was performed under the following conditions: 16°C for 30 min, 42°C for 30 min, and 85°C for 5 min with a sample volume of 15 μl. The real-time PCR was performed in the 7500 Real-Time PCR system (ABI, Applied Biosystems, USA) using TaqMan MicroRNA Assays and TaqMan Universal PCR Master Mix with a sample volume of 20 μl (7.67 μL nuclease-free water, 10 μl TaqMan Universal PCR Master Mix, 1 μl TaqMan Small RNA Assay, and 1.33 μl product from the reverse transcription reaction). All reagents used in this study were purchased from Thermo Fisher Scientific Inc. (Thermo Scientific/Ambion, USA). All the samples were run in triplicate, and no-template controls were tested alongside actual samples in each experiment. After initial enzyme activation at 95°C for 10 min and 40 cycles of 15 s denaturation at 95°C were performed, 1 min annealing and extension at 60°C was carried out. Raw RT-qPCR data were obtained using Applied Biosystems7500 Real-Time PCR Software version 2.0, and the cycle threshold (Ct) values were used to analyze the expression levels of targeted miRNAs. The expression levels were normalized to the RNU6B (endogenous control) expression. The miRNA relative amounts were determined using the comparative Ct method (ΔΔCT = ΔCT [the tumor tissue sample] – ΔCT [the corresponding non-neoplastic squamous epithelium]). The fold changes in the expression of the three miRNAs between each tumor sample and its corresponding non-neoplastic squamous epithelium were determined using the the 2–ΔΔCT method.

### Statistical Analysis

Data obtained in the study were analyzed statistically using SPSS version 20.0 software. Fold changes for miRNAs were expressed graphically and numerically. Comparisons among primary localizations were performed for Log2 transformed miRNA expression levels using one-way ANOVA and Tukey’s honest significant difference tests (Tukey’s HSD). Individual and combined Receiver Operator Characteristics (ROC) curve analyses for miRNAs with significant differences among SCC groups were performed to assess the diagnostic accuracy. The statistical confidence level was set at 0.95 (α = 0.05). Post hoc power analysis was performed for miRNA levels with a statically insignificant difference using G*Power version 3.1.9.2. Differences in expression profiles of miR-126 and mir-486-5p among groups were significant, with powers of 0.95 and 0.93, respectively

## RESULTS

**Figure 1:**
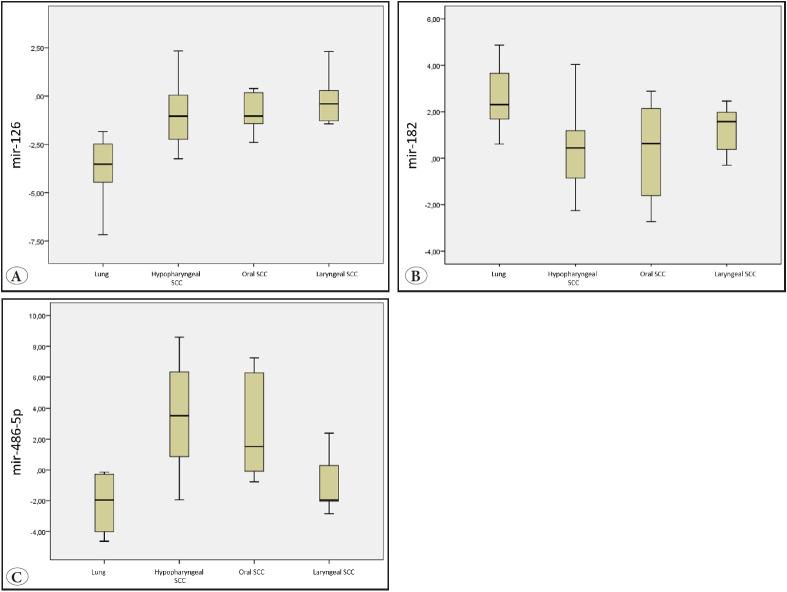
**A-C)** Expression levels of the five candidate miRNAs in lung, oral, hypopharyngeal and laryngeal SCCs. A; mir-126, B; mir-182, C; mir-486-5p. Scale at y-axis represents fold change values normalized to the corresponding squamous epithelium. Line inside the box: median, box: interval between the 25th and 75th percentiles, whiskers: interval between the 10th and 90th percentiles, circles: outliers.

The SCC samples and corresponding non-neoplastic squamous epithelia of 40 patients were analyzed for mir-126, mir-182 and mir-486-5p via RT-qPCR analysis. One lung SCC and one laryngeal SCC patient were excluded from the study because no expression for RNU6b was detected. In an attempt to identify anatomical site-specific miRNA expression patterns, the relative expression levels of these miRNAs in the SCC subgroups were determined and log2 transformed, as represented in the box plot graphs (Figure 1). The results shown in Figure 1 indicated that mir-126 was under-expressed in all groups. mir-182 was overexpressed in lung and laryngeal SCCs and underexpressed in oral and hypopharyngeal SCCs. mir-486-5p was overexpressed in oral and hypopharyngeal SCCs and underexpressed in lung and laryngeal SCCs.

There were no statistically significant differences in the mir-126, mir-182 and mir-486-5p expression profiles between keratinizing type SCCs and non-keratinizing type SCCs in all anatomic localizations (p>0.05). The miRNA expression profiles in the lung SCCs were compared with the expression profiles in the oral cavity, laryngeal, and hypopharyngeal SCCs. The results are summarized in Table 2. The findings showed that there were noticeably different expression levels for mir-126, mir-182, and mir-486-5p between the lung SCCs and hypopharyngeal SCCs (p < 0.05); for mir-126, mir-182, and mir-486-5p between the lung SCCs and oral SCCs (p < 0.05); and for mir-126 between the lung SCCs and laryngeal SCCs (p < 0.05).

**Table 2:** Analysis of variance (ANOVA) results for miRNA expression between lung, oral, hypopharyngeal, and laryngeal SCC.

	**Lung SCC**			**Oral SCC**			**Hypopharyngeal SCC**			**Laryngeal SCC**			
**miRNA**	n	mean	sd	n	mean	sd	n	mean	sd	n	mean	sd	p^§^
**mir-126**	9	-3.75	1.64	10	-0.91	0.98	10	-0.98	1.59	9	-0.04	1.40	<0.001**
**mir-182**	9	2.70	1.41	10	0.49	1.99	10	0.40	1.94	9	1.21	1.11	0.024*
**mir-486-5p**	9	-2.25	1.88	10	2.85	3.19	10	3.65	3.26	9	-0.50	3.96	<0.001**

**SCC:** Squamous cell carcinoma, *Statistically significant at the 0.95 confidence level. **Statistically significant at the 0.999 confidence level. ^§^One-way ANOVA was used for comparison

**Figure 2:**
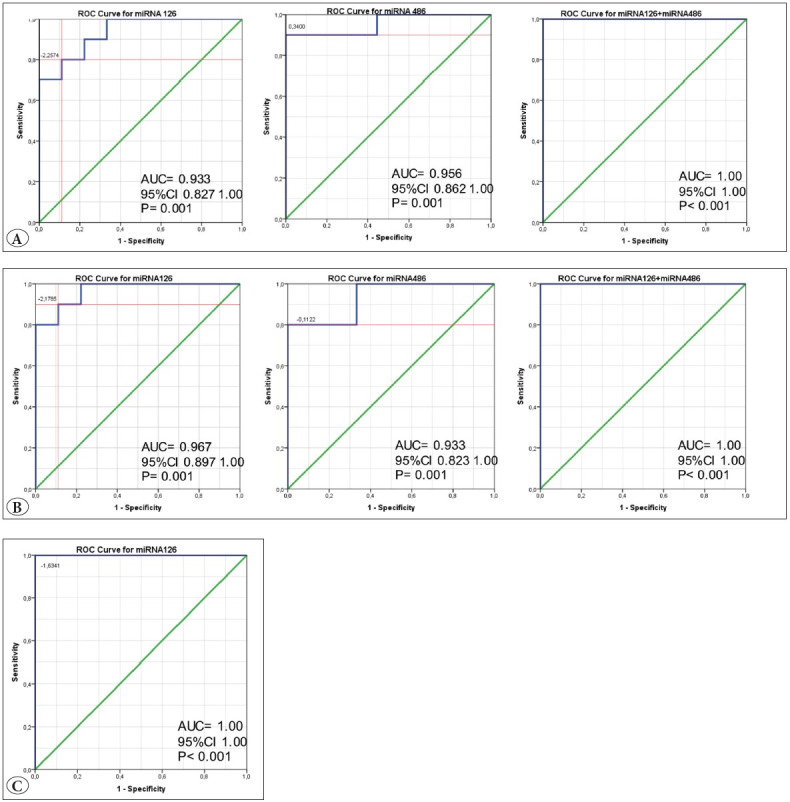
**A)** ROC curve analysis of the mir-126, mir486-5p and combination of mir-126 and mir-486-5p signature for differentiating lung SCCs from hypopharyngeal SCCs; mir- 126 revealed 80.0% specificity and 88.0% sensitivity with the cut-off point was -2.257 and mir-486-5p revealed 90.0% specificity and 100.0% sensitivity with the cut-off point was 0.34. Combined expression profiles of mir-126 and mir-486-5p revealed an AUC of 1.00. **B)** Analysis of the mir-126, mir486-5p and combination of mir-126 and mir-486- 5p signature for differentiating lung SCCs from oral SCCs; mir-126 revealed 90.0% specificity and 88.0% sensitivity with the cut-off point was-2.1785 and mir-486-5p revealed 80.0% specificity and 100.0% sensitivity with the cut-off point was -0.1122. Combined expression profiles of mir-126 and mir-486-5p revealed an AUC of 1.00 (100% sensitivity and 100% specificity). **C)** Analysis of the mir-126 signature for differentiating lung SCCs from laryngeal SCCs; mir-126 revealed 100.0% specificity and 100.0% sensitivity with the cut-off point was-1.6341. ***ROC:*** *Receiver operating characteristic,* ***AUC:*** *Area under the curve,* ***CI:*** *Confidence interval*

ROC curve analysis was performed to assess the value of these miRNA expression profiles as specific molecular signatures for anatomic localization. The area under the curves (AUC) for mir-126, mir-182, and mir-486-5p were 0.93, 0.84, and 0.95, respectively (p < 0.05). All three miRNAs had good discriminative power in differentiating lung SCCs from hypopharyngeal SCCs. The combination signatures of mir-126 and mir-486-5p showed better prediction than individual miRNA. The optimal cut-off values of mir-126 and mir-486-5p were – 2.25 and 0.34, respectively. These findings showed that the combined expression profiles of mir-126 and mir-486-5p could distinguish lung SCCs from hypopharyngeal SCCs with 100% sensitivity and 100% specificity (Figure 2).

mir-126 and mir-486-5p were differentially expressed between the lung SCCs and oral SCCs. ROC curve analysis indicated that these miRNA expression profiles were a significant discriminant factor between lung SCCs and oral SCCs. The AUCs for mir-126 and mir-486 were 0.93 and 0.96, respectively, and when combined, the AUC was 1.00 (p < 0.05). The optimal cut-off values of mir-126 and mir-486 were – 2.17 and – 0.11, respectively. In addition to the hypopharyngeal SCC, the combined expression profiles of mir-126 and mir-486 could distinguish lung SCCs from oral SCCs with 100% sensitivity and 100% specificity (Figure 2).

Only mir-126 was differentially expressed between lung SCCs and laryngeal SCCs. ROC curve analyses revealed that the optimal cut-off value of mir-126 was – 1.63, and mir-126 was sufficiently effective to differentiate between lung SCCs and laryngeal SCCs (AUC = 1.00) (Figure 2). The laryngeal SCCs group included five glottic laryngeal SCC cases and four supraglottic laryngeal SCC cases that predominantly located in the epiglottis without false cord involvement. There were no statistically significant differences in the mir-126 expression profile between the supraglottic laryngeal SCCs and the glottic laryngeal SCCs cases. (p > 0.05).

## DISCUSSION

Oral and hypopharyngeal SCCs originate from squamous cells in the mucosal lining of the upper aerodigestive tract (1). At the third month of prenatal development, the larynx is lined entirely by pseudostratified epithelium (30). With age, this epithelium is gradually replaced by stratified squamous epithelium except for the ventricle, the subglottic, and rare microscopic patches in the supraglottic region (30,31). The glottic and some of the supraglottic laryngeal SCCs arises from the stratified squamous epithelium. However, the rest of the laryngeal SCCs arises from the metaplastic squamous epithelium (32,33).

Lung SCCs originate in the lower respiratory tract epithelium, and unlike the upper aerodigestive tract, this epithelium does not normally contain squamous cells. Long-term exposure to irritants causes epithelial changes in the bronchial epithelium, creating a reparative reaction that causes squamous metaplasia and epithelial dysplasia (34,35). Lung SCCs develop from these metaplastic-dysplastic cells (34). SCCs arising from keratinocytes or metaplastic cells often exhibit similar histological appearance under the light microscope.

Considering the differences in the developmental stages of these tumors, SCCs may differ in terms of genetic and epigenetic features. Many studies have shown that tumors with similar histological appearance originating from different anatomic localizations have genetic and epigenetic differences specific to the localization (8,10,34).

Studies have also emphasized that tumors carry specific genetic and epigenetic features to metastatic foci and the localization of tumors can be predicted by evaluating these properties (14,15). In this study, the expression patterns of mir-126, mir-182 and mir-486-5p were analyzed in lung, oral cavity, hypopharyngeal, and laryngeal SCCs, which have been shown to be highly significant and consistently dysregulated miRNAs in lung cancer. The study findings revealed that the lung, oral cavity, hypopharyngeal, and laryngeal SCCs had distinct miRNA expression profiles. Munoz-Largacha et al. also showed that lung SCCs and HNSCCs had distinct miRNA expression profiles (10) in a study which demonstrated that 48 miRNAs were differentially expressed between lung SCCs and HNSCCs. They found mir-10a expression was greater (-3.5 to - 5.3-fold) in lung SCCs and miR-10b expression was greater (1.7-fold) in HNSCCs; the expression ratio of mir-10a to mir-10b had a strong predictive power of tumor anatomical site in the training and the validation data sets (AUC: 0.92-0.98). They also stated that expression profile of several miRNA may be useful for discriminating between HNSCC and lung SCC. The fold change of mir-126, mir-182 and mir-486 between HNSCC and lung SCC were – 1.63, 1.03 and 1.33, respectively, in their data sets. In addition to those findings, from the current study results it was observed that mir-126, mir-182, and mir-486-5p were differentially expressed between the lung SCCs and hypopharyngeal SCCs, mir-126 and mir-486-5p were differentially expressed between the lung SCCs and oral SCCs, and mir-126 was differentially expressed between the lung SCCs and laryngeal SCCs. The most important findings of this study were that the expression profiles of mir-126 and mir-486-5p were strongly correlated with tumor localization.

mir-126 has a role in the regulation of 81 genes such as MAPK1, VEGFA, PIK3CA, PIK3CD, AKT1, and STK1119. Target pathway prediction has shown that most of these genes are involved in the regulation of proliferation (36,37). mir-126 plays an important role in lung tumorigenesis by influencing the PI3K/Akt pathway and MAPK signaling pathway via regulation of AKT1, PIK3CA, and MAPK1 (36,38). Crawford et al. also observed that alteration of mir-126 expression affected adhesion, and the migratory and invasive capacity of lung cancer, through Crk regulation (39). Several studies have indicated that mir-126 is significantly downregulated miRNA in lung cancer (17,20,39). The current study findings showed that although mir-126 was downregulated in all SCC groups, the expression of mir-126 was significantly lower in lung SCCs than in other SCCs.

Studies have indicated that mir-126 is also an important biomarker in the diagnosis of lung cancer (17,20,21,38,39). Song et al. revealed that combinations of mir-126, mir-182, and mir-205 have good accuracy in the prediction of lung carcinoma (21). Zhu et al. and Sanfienzo et al. reported that analysis of serum and plasma miRNAs expression profiles, including mir-126, could distinguish lung carcinoma patients from healthy volunteers (22,40). Furthermore, Barshack et al. compared the mir-126 expression profile of lung tumors and metastases of the tumors to the lungs, and observed that mir-126 expression was downregulated in primary lung tumor and expression analyses could distinguish primary lung tumor from lung metastases (20). The current study findings showed that mir-126 expression profile was highly specific for tumor anatomic localization and analysis of expression levels proved to be a promising method with the potential to differentiate between lung SCCs and laryngeal SCCs (p < 0.0001, 95% CI).

mir-486-5p is another important miRNA, involved in lung tumorigenesis, which induces translation of numerous validated genes, such as PTEN, CDK4, ARHGAP, and PIK3R1 (36,37). The interaction between mir-486-5p and these genes influences cell cycle progression, apoptosis, and the PI3K/Akt pathway (36,37). Yu et al. showed that mir-486-5p inhibited the development and invasion of lung tumors through the repression of GAB2, while Wang et al. showed that the downregulation of miR-486-5p contributed to the development of lung cancer by regulating ARHGAP5 (23,24). Several studies have shown that mir-486-5p acts as a tumor suppressor and is one of the most significantly downregulated miRNAs in lung cancer (18,23,24). mir-486-5p could also provide a diagnostic approach for detecting lung cancer (23,24). In meta-analyses by Tian et al., it was indicated that mir-486 expression analyses of tissue, plasma, blood, and serum could provide a biomarker for lung cancer diagnosis (18,25). In addition to this result, the current study findings suggested that mir-486-5p expression levels were also highly specific for tumor localization and mir486-5p has great potential to be a novel, sensitive, and reliable biomarker for differential diagnosis. It was determined that mir-486-5p was down-regulated in lung SCCs and laryngeal SCCs, and up-regulated in hypopharyngeal and oral cavity SCCs. The analysis of mir-486-5p expression profiles can differentiate lung SCCs from hypopharyngeal SCCs with 90% sensitivity and 100% specificity and can differentiate lung SCCs from oral SCCs with 80% sensitivity and 100% specificity. In addition, the combined expression profiles of mir-126 and mir-486-5p could distinguish lung SCCs from hypopharyngeal SCCs and oral SCCs with 100% sensitivity and 100% specificity.

mir-182 can affect 2105 genes and plays an important role in carcinogenesis in various cancer tissues (36,37). It has been reported that the target genes of mir-182-5p are enriched in 42 KEGG pathways such as ‘NSCLC’, ‘cell cycle’, ‘apoptosis’, ‘p53 signaling pathway’, and ‘Wnt signaling pathway’ (37). Lou et al. analyzed the TCGA database and observed that mir-182 regulated 81 gene functions in lung SCCs by repressing these gene functions (19). In addition, analyses of TCGA data have revealed that mir-182, whose over expression has been reported recently to be associated with overall poor survival in patients with lung SCC, was one of the most upregulated miRNAs in lung SCCs (19,41). Compatible with this research, the current study results showed that mir-182 was upregulated in the oral, hypopharyngeal, laryngeal, and lung SCC.

The results of this study demonstrated that SCCs arising from different anatomic localizations in the oral, hypopharyngeal, laryngeal, and lung regions had mir-126 and mir486-5p expression profiles specific to the localization and these expression profiles were strongly associated with tumor localization. The weakest point of this study was the small sample size. Nevertheless, the findings of this study will provide a basis for further investigations into miRNA-based differential diagnosis. Further research of larger samples may help to clarify the diagnostic utility of these miRNAs as a predictive tool.

In conclusion, recent studies have revealed that the miRNAs expressed differentially between different tissue and tumor types and their highly specific expression profiles can be used for diagnosis through the classification of primary cancers and their metastases. The results of this study revealed that the SCCs arising from the oral, hypopharyngeal, laryngeal, and lung regions had distinct mir-126 and mir-486-5p expression profiles, and mir-126 and mir-486-5p expression analysis may provide potential biological markers for the prediction of the primary localization of SCCs.

## Conflict of INTEREST

The authors declare that they have no conflict of interest.

## FUNDING

This study was supported by the Gulhane Military Medical Academy, Haydarpasa Training Hospital Epidemiology Committee.
